# Factors Associated with All-Cause 30-Day Mortality in Indonesian Inpatient COVID-19 Patients at Cipto Mangunkusumo National General Hospital

**DOI:** 10.3390/jcm13102998

**Published:** 2024-05-20

**Authors:** Ikhwan Rinaldi, Mira Yulianti, Evy Yunihastuti, Wulyo Rajabto, Cosphiadi Irawan, Lugyanti Sukrisman, Andhika Rachman, Nadia Ayu Mulansari, Anna Mira Lubis, Findy Prasetyawaty, Rahmat Cahyanur, Dimas Priantono, Ardhi Rahman Ahani, Abdul Muthalib, Aru Sudoyo, Tubagus Djumhana Atmakusuma, Arry Harryanto Reksodiputro, Zubairi Djoerban, Karmel Tambunan, Kevin Winston, Yuli Maulidiya Shufiyani, Lowilius Wiyono, Samuel Pratama, Brenda Cristie Edina

**Affiliations:** 1 Hematology and Medical Oncology, Internal Medicine Department, Cipto Mangunkusumo National General Hospital, Faculty of Medicine, Universitas Indonesia, Jakarta 10430, Indonesia; wulyo02@gmail.com (W.R.); cosphiadi@gmail.com (C.I.); lugyanti@gmail.com (L.S.); andhikarachman@gmail.com (A.R.); mirafanani@gmail.com (A.M.L.); findyprasetyawaty@gmail.com (F.P.); rahmat.cahyanur@gmail.com (R.C.); dimas.priantono@gmail.com (D.P.); ardhi.ahani@gmail.com (A.R.A.); amuthalib@hotmail.com (A.M.); arusudoyo@gmail.com (A.S.); endjum@hotmail.com (T.D.A.); arryrek@gmail.com (A.H.R.); zubairi_djoerban@yahoo.com (Z.D.); karmeltambunan@yahoo.co.id (K.T.); 2 Respirology and Critical Care, Internal Medicine Department, Cipto Mangunkusumo National General Hospital, Faculty of Medicine, Universitas Indonesia, Jakarta 10430, Indonesia; mirayulianti.md@gmail.com; 3 Allergy and Immunology, Internal Medicine Department, Cipto Mangunkusumo National General Hospital, Faculty of Medicine, Universitas Indonesia, Jakarta 10430, Indonesia; evy.yunihastuti@gmail.com; 4 Faculty of Medicine, Universitas Indonesia, Jakarta 10430, Indonesia; kevinwinston4@gmail.com (K.W.); yulimaulidiya.ym@gmail.com (Y.M.S.); lowilius.wiyono@alumni.ui.ac.id (L.W.); samuel.pratama@gmail.com (S.P.); brenda.cristie@ui.ac.id (B.C.E.)

**Keywords:** COVID-19, mortality, cancer, D-Dimer

## Abstract

**Introduction**: Indonesia, as a developing country, has limited data on the factors associated with 30-day mortality in COVID-19 patients in Indonesia. As a matter of fact, study analyzing factors associated with 30-day mortality of COVID-19 infection in Indonesia has never been conducted. This study aims to fill this gap in the literature by conducting a large-scale analysis of factors associated with 30-day mortality in COVID-19 patients in Indonesia. **Method**: This study employed a single-center retrospective cohort observational design, and was conducted at Cipto Mangunkusumo National General Hospital between the years 2022 and 2023. Sampling was conducted using the consecutive sampling method. The study included patients aged 18 years and above who had been confirmed to have COVID-19 infection. Survival analysis was conducted using Kaplan–Meier and multivariate Cox regression analysis. **Result**: Our study included a total of 644 patients, with 120 patients (18.6%) expiring within 30 days. In the multivariate analysis using the backward Wald method, severe COVID-19 (HR: 7.024; 95% CI: 3.971–12.744; *p* value: <0.0001), moderate COVID-19 infection (HR: 1.660; 95% CI: 1.048–2.629; *p* value: 0.031), liver cirrhosis (HR: 3.422; 95% CI: 1.208–9.691; *p* value: 0.021), female sex (HR: 1.738; 95% CI: 1.187–2.545; *p* value: 0.004), old age (HR: 2.139; 95% CI: 1.279–3.577; *p* value: 0.004), high leukocyte (HR: 11.502; 95% CI: 1.523–86.874; *p* value: 0.018), high NLR (HR: 1.720; 95% CI: 1.049–2.819; *p* value: 0.032), high CRP (HR: 1.906; 95% CI: 1.092–3.329; *p* value: 0.023), high procalcitonin (HR: 3.281; 95% CI: 1.780–6.049; *p* value: 0.001), and high creatinine (HR: 1.863; 95% CI: 1.240–2.800; *p* value: 0.003) were associated with 30-day mortality from COVID-19 infection. Subgroup analysis excluding cancer patients showed that age, D-Dimer, CRP, and PCT were associated with 30-day mortality in COVID-19 patients, while steroid therapy is protective. **Conclusions**: This study finds that COVID-19 severity, liver cirrhosis, sex, age, leukocyte, NLR, CRP, creatinine, and procalcitonin were associated with COVID-19 mortality within 30 days. These findings underscore the multifactorial nature of COVID-19 infection mortality. It is important, therefore, that patients which exhibit these factors should be treated more aggressively to prevent mortality.

## 1. Introduction

As is now widely known, the COVID-19 virus, also known as SARS-CoV-2, has emerged as one of the most significant global health challenges of the 21st century. The virus was first identified in December 2019 in the city of Wuhan, China. This virus then quickly spread worldwide, leading to a pandemic declaration by the World Health Organization (WHO) in March 2020. The unprecedented scale and impact of COVID-19 have reshaped daily life, economies, and public health strategies across the globe, with over 6 million deaths and millions more people infected [1,2,3].

During the pandemic, the total number of patients requiring hospitalization increased sharply, and the demand for intensive care unit (ICU) beds increased during the pandemic [4]. This has put a strain on the healthcare system and, at that time, it was difficult to provide adequate care to all patients.

Mortality from COVID-19 is a major concern for healthcare providers. There are many risk factors of mortality from COVID-19. One of the risk factors is age, which has consistently emerged as one of the most significant risk factors for COVID-19 mortality [5]. Multiple studies have shown that older individuals face a substantially higher risk of death from COVID-19 compared to younger age groups [6,7,8]. Older age is linked to a weakening of the immune system and an increased occurrence of other health issues like heart disease, diabetes, and respiratory ailments. These factors raise the chances of experiencing severe outcomes from COVID-19 [9].

There are other risk factors such as BMI. Studies have consistently demonstrated a dose–response relationship between BMI and mortality risk, with higher BMI categories associated with increased odds of severe outcomes, including death [10,11]. Comorbidities appear to also be linked with COVID-19 mortality [12,13].

D-Dimer, a biomarker indicative of blood clot formation and breakdown, has been shown to be useful in predicting prognosis of COVID-19 patients. Elevated levels of D-Dimer have been consistently associated with an increased risk of mortality [14,15]. COVID-19 triggers a hyperinflammatory response and a propensity for thrombotic events, leading to microvascular damage and organ dysfunction.

Indonesia is a developing country with suboptimal healthcare. Furthermore, there are many barriers and challenges in conducting research in Indonesia, such as the low priority of health research and poor research culture [16]. This has resulted in limited publications in developing countries, including Indonesia. Thus, even post-pandemic, Indonesia still has no data from study on the factors associated with 30-day mortality of COVID-19 patients in the country.

Other developing countries have more established data on COVID-19. For example, a study by Ismail et al. in Malaysia demonstrated that older age, male sex, and multiple comorbidities were factors associated with COVID-19 mortality [17]. Meanwhile, a study in Thailand showed that older age, the use of high flow nasal cannula, the use of mechanical ventilation, and hydrocortisone treatment were associated with in-hospital mortality [18].

Thus, this study aims to fill this gap in the literature by conducting a large-scale analysis of factors associated with 30-day mortality in COVID-19 patients in Indonesia.

## 2. Method

### 2.1. Study Design

This study employed a single-center retrospective cohort observational design, and was conducted at Cipto Mangunkusumo National General Hospital between the years 2022 and 2023. The hospital is a national tertiary referral hospital in Indonesia. Data for this study were sourced from patient medical records covering the period from January 2020 to July 2023.

The dependent variable for this study was 30-day mortality. Meanwhile, the independent variables were the severity of the COVID-19 infection, sex, age, BMI, hemoglobin levels, leukocyte count, platelet count, neutrophil-lymphocyte ratio (NLR), platelet-lymphocyte ratio, C-reactive protein (CRP) levels, D-Dimer, pro-calcitonin levels, aspartate aminotransferase (AST) levels, alanine aminotransferase (ALT) levels, creatinine levels, steroid therapy, and comorbidities. Assessed comorbidities were cancer, hypertension, diabetes, chronic kidney disease, liver cirrhosis, and cardiovascular diseases.

### 2.2. Operational Definitions

COVID-19 infection status was determined using a PCR test and/or antigen test. The severity of COVID-19 was categorized into three groups: mild, moderate, and severe, following the guidelines provided by the Indonesian COVID-19 management (edition 4). Survival time was calculated from the day of hospital admission until the patient expired or survived. If the patient was lost to follow-up, such as discharge against medical advice (DAMA), the latest day of follow-up would be used, and the patient would be classified as censored in survival analysis.

Age was determined from the date of birth. Sex was determined using biological sex. BMI classification for this study used criteria for Asia–Pacific, which are: (1) <18.5 kg/m^2^ as underweight; (2) 18.5 to 22.9 kg/m^2^ as normal weight; (3) 23.0 to 24.9 kg/m^2^ as overweight; and (4) ≥25 kg/m^2^ as obese [19].

Laboratory parameters were collected either from the first day of hospital admission or the closest subsequent day, while comorbidities were identified through examination of the diagnoses on the medical records. All laboratory parameters were conducted at Cipto Mangunkusumo National General Hospital. The variable of cardiovascular disease included stroke, heart failure, a history of myocardial infarction, arrythmia, and peripheral artery disease. Meanwhile, the variable of cancer included all types of cancer, irrespective of the cancer stage.

### 2.3. Participants and Inclusion Criteria

The study included patients aged 18 years and above who had been confirmed to have a COVID-19 infection. Patients with incomplete medical record data were excluded from the study. Sampling was conducted using consecutive sampling.

### 2.4. Statistical Analysis

Descriptive analysis was conducted to summarize the characteristics of patients. Numerical data with a normal distribution were described using the mean and standard deviation, while numerical data with a non-normal distribution were presented using the median and minimum–maximum values. Categorical data were presented using percentages.

Comparisons between variables were conducted using a chi-square for categorical data and a *t*-test or Mann–Whitney test for numerical data, depending on the data normality.

Survival analysis was conducted using Kaplan–Meier analysis and Cox regression. For survival analysis, numerical variables were converted into categorical variables using median/mean as the cutoff, or using predetermined cutoffs. For the Kaplan–Meier analysis, a Log-rank test and Breslow tests were used to analyze the statistical differences in survival.

Bivariate Cox regression for factors associated with 30-day mortality was presented as a hazard ratio (HR) with 95% confidence intervals. Multivariate Cox regression was conducted using a backward Wald method.

All collected data were processed using SPSS version 26.0.

### 2.5. Ethical Approval

This study was approved by ethical committee of Cipto Mangukusumo National General Hospital, with approval number KET-300/UN2.F1/ETIK/PPM.00.02/2022. As this study used secondary data, no consent was determined to be required by ethical committee of Cipto Mangukusumo National General Hospital.

## 3. Results

Our study included a total of 644 patients hospitalized with COVID-19 infection. Their characteristics are presented in Table 1. Within our study population, 120 patients (18.6%) expired within 30 days of hospitalization. Mild, moderate, and severe COVID-19 infections constitute 35.9%, 58.9%, and 5.3% of total patients, respectively.

A total of 281 (43.6%) patients were males, and 363 (56.4%) were females. The mean age of the patients was 48.75 years, and the group that expired had statistically older patients than the group that survived (mean 53.83 vs. 47.57 years; *p* value: <0.0001). The group that expired also had higher proportion of cancer (65% vs. 34.7%; *p* value: <0.0001). Furthermore, there are differences in the proportions of patients that survived and expired, as determined by the chi-square tests, on COVID-19 severity (*p* value: <0.0001), BMI (*p* value: 0.002), chronic kidney disease (*p* value: <0.0001), and steroid use (*p* value: 0.004).

For the numerical variables, the patients that expired also had lower BMI (*p* value: <0.0001), lower hemoglobin (*p* value: <0.0001), lower hematocrit (*p* value: <0.0001), lower platelet (*p* value: <0.0001), and higher AST (*p* value: 0.033).

**Figure 1:**
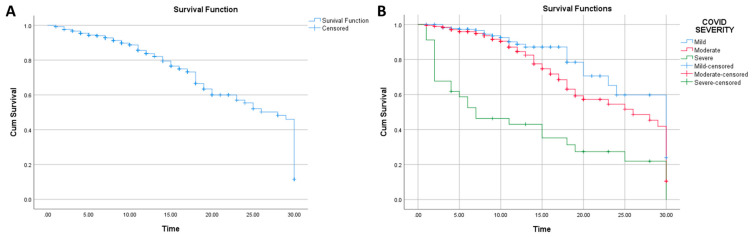
(**A**): Kaplan–Meier analysis for survival (n: 644); (**B**): Kaplan–Meier analysis for survival based on COVID-19 severity (n: 644).

The median survival assessed by Kaplan–Meier analysis was 28 days (95% CI: 26.594–29.406), while the mean survival was 22.643 days (95% CI: 21.442–23.843) (Figure 1). Analyses conducted showed that COVID-19 severity was associated with 30-day survival (Log Rank *p* value: <0.0001; Breslow *p* value: <0.0001) (Figure 1).

In Figure 1, it can be seen that the highest survival was from the group with a mild COVID-19 infection, as the survival from moderate and severe COVID-19 were lower. Noteworthy, from the figure, it appears that the group with a severe COVID-19 infection has a high proportion of patients expiring in less than 10 days when compared with other groups.

**Figure 2:**
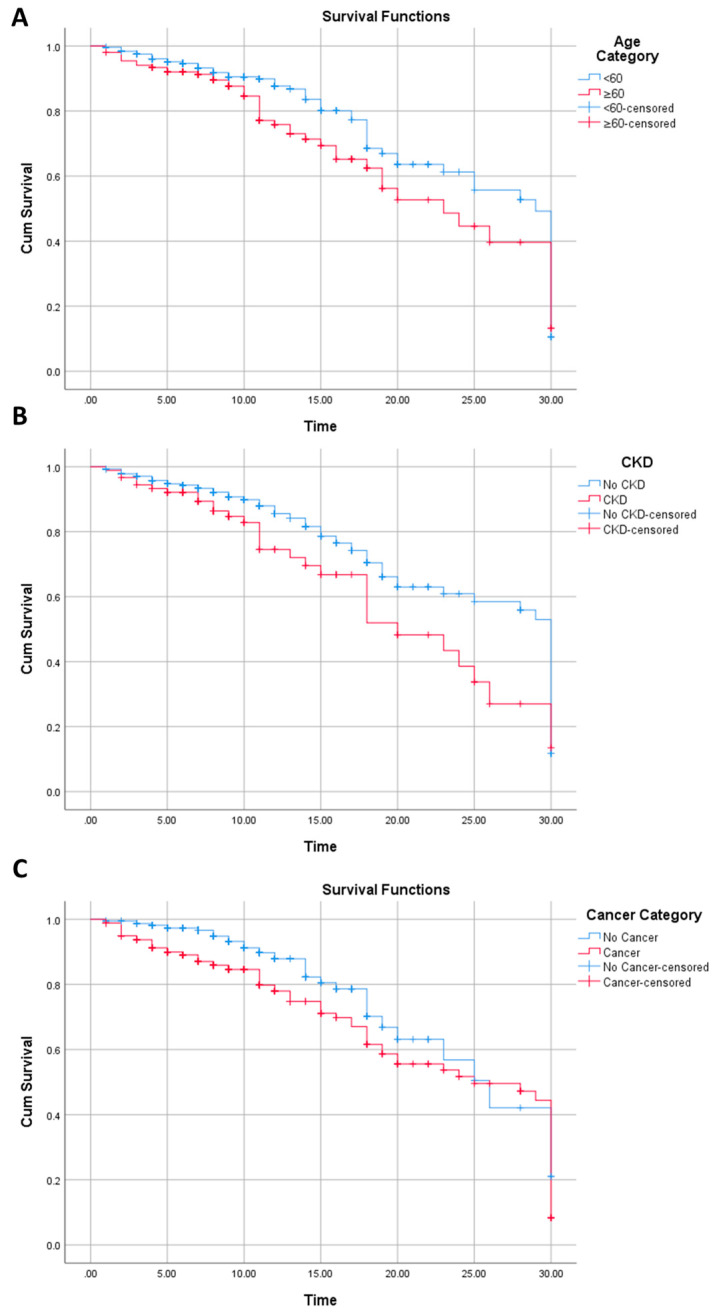
Kaplan–Meier analysis for survival on age (**A**), chronic kidney disease (**B**), and cancer (**C**).

Similarly, chronic kidney disease (Log Rank *p* value: 0.011; Breslow *p* value: 0.047), age (Log Rank *p* value: 0.030; Breslow *p* value: 0.042), cancer status (Log Rank *p* value: 0.003; Breslow *p* value: <0.0001), hemoglobin (Log Rank *p* value: 0.051; Breslow *p* value: 0.002), leukocyte (Log Rank *p* value: <0.0001; Breslow *p* value: <0.0001), NLR (Log Rank *p* value: <0.0001; Breslow *p* value: <0.0001), D-Dimer (Log Rank *p* value: 0.006; Breslow *p* value: 0.001), CRP (Log Rank *p* value: <0.0001; Breslow *p* value: <0.0001), PCT (Log Rank *p* value: <0.0001; Breslow *p* value: <0.0001), AST (Log Rank *p* value: 0.001; Breslow *p* value: 0.007), and creatinine level (Log Rank *p* value: 0.002; Breslow *p* value: 0.002) were variables associated with survival in the Kaplan–Meier analysis (Figure 2).

Per Figure 2, patients with an age of <60 years old had higher survival than those with an age of ≥60 years old from the start of the follow-up to the last follow-up. This is similar with the chronic kidney disease variable. In contrast, the survival of cancer patients was generally lower than non-cancer patients, but only until approximately 25 days of follow-up, where, subsequent to this, the survival of cancer patients is slightly higher than non-cancer patients.

Cox regression results are presented in Table 2. In the bivariate Cox regression analysis, it was found that severe COVID-19 infection, chronic kidney disease, cancer status, age, hematocrit, leukocyte, NLR, D-Dimer, CRP, procalcitonin, and AST were associated with 30-day COVID-19 mortality.

In the multivariate analysis using the backward Wald method, only nine variables were found significantly associated with COVID-19 mortality. From these nine variables, severe COVID-19 infection had the highest HR (HR: 7.024; 95% CI: 3.971–12.744; *p* value: <0.0001). Meanwhile, moderate COVID-19 infection had a lower HR (HR: 1.660; 95% CI: 1.048–2.629; *p* value: 0.031).

Other variables associated with COVID-19 mortality include liver cirrhosis (HR: 3.422; 95% CI: 1.208–9.691; *p* value: 0.021), sex (HR: 1.738; 95% CI: 1.187–2.545; *p* value: 0.004), age (HR: 2.139; 95% CI: 1.279–3.577; *p* value: 0.004), leukocyte (HR: 11.502; 95% CI: 1.523–86.874; *p* value: 0.018), NLR (HR: 1.720; 95% CI: 1.049–2.819; *p* value: 0.032), CRP (HR: 1.906; 95% CI: 1.092–3.329; *p* value: 0.023), procalcitonin (HR: 3.281; 95% CI: 1.780–6.049; *p* value: 0.001), and creatinine (HR: 1.863; 95% CI: 1.240–2.800; *p* value: 0.003).

A subgroup analysis excluding cancer patients was conducted to address the potential confounding factors related to cancer, as cancer is known to cause elevated NLR, D-Dimer, CRP, and PCT (Table 3).

In this subgroup analysis, the multivariate analysis revealed that age (HR: 2.225; 95% CI: 1.184–4.182; *p* value: 0.013), D-Dimer (HR: 2.942; 95% CI: 1.126–7.684; *p* value: 0.028), CRP(HR: 4.356; 95% CI: 1.665–11.398; *p* value: 0.003), PCT (HR: 8.098; 95% CI: 1.913–34.278; *p* value: 0.004), were associated with 30-day COVID-19 mortality. Meanwhile, steroid use showed a protective effect against COVID-19 mortality (HR: 0.332; 95% CI:0.154–0.714; *p* value: 0.005).

## 4. Discussion

This study was the first to investigate the factors associated with 30-day mortality in COVID-19 patients in Indonesia. This study found that the severity of the COVID-19 infection is a major risk factor for 30-day mortality, similar to other studies [20,21,22]. Also, this finding is similar to other infections, such as pneumonia, sepsis, and meningitis, where the more severe the infection is, the higher the likelihood for complications and mortality. Therefore, it is important to identify patients with severe COVID-19 infection early on, so that they can receive the appropriate treatment.

This study also found that an age of ≥60 years is a significant risk factor for 30-day mortality (HR: 2.139). Older patients are more likely to have underlying health conditions that make them more susceptible to the complications of COVID-19 [6,23,24]. The other main factors for COVID-19 mortality in the elderly are immunosenescence and chronic inflammation [25,26].

A study conducted by Doerre et al. showed that male patients were more likely to die from COVID-19 than female patients [27]. The reasons for this are not fully understood, but it may be due to the fact males might have higher exposure to COVID-19, or have a higher likelihood of smoking [28]. In contrast, we found that female patients had a higher hazard ratio for mortality. It is possible that the female sex is related to poorer nutrition in Indonesia.

This study also showed that liver cirrhosis was associated with 30-day COVID-19 morality. Liver cirrhosis is a common chronic condition, especially in developed countries, characterized by scarring of the liver tissue, often resulting from long-term liver damage and inflammation, such as from long-term alcohol drinking or obesity. COVID-19 infection primarily affects the respiratory system, but can also have systemic effects, including on the liver. Liver cirrhosis often leads to immune system dysfunction, making individuals more susceptible to infections, including viral infections like COVID-19 [29]. A study by Wang et al. showed that liver cirrhosis is an independent predictor of COVID-19 mortality [30].

Finally, this study found that leukocytosis, high NLR, high CRP, and high PCT are all associated with 30-day mortality. These are all markers of inflammation, and it is widely known that they are indicative of more severe immune responses to COVID-19 and general infections [31,32,33].

In the multivariate analysis, D-Dimer was not found to be statistically significant, as it is confounded by cancers, which also increase D-Dimer. When a subgroup analysis was conducted excluding cancer patients, D-Dimer became statistically significant. However, there are still other factors that can influence D-Dimer other than cancer, such as diabetes, age, and various comorbidities.

D-Dimer is a fibrin degradation product that is elevated in various clinical conditions, including COVID-19. A study conducted by Ali et al. discovered that high D-Dimer levels were independently correlated with the need for invasive mechanical ventilation (IMV) in COVID-19 patients [34]. The study also found that low levels of D-Dimer could predict post-IMV survival of mechanically ventilated COVID-19 patients. Another study by Zuckier et al. found that increased levels of D-Dimer were associated with pulmonary embolism in COVID-19 patients [35]. Furthermore, a study by Wang et al. found that persistent elevation of D-Dimer levels following recovery from COVID-19 was associated with an increased risk of thrombotic events [36].

Cancers cause derailment of the immune system and inflammation in patients [37]. Cancer patients infected with COVID-19 face a significantly higher risk of severe illness and death compared to the general population. Meta-analyses by ElGohary et al. estimated an approximately 3.23 odds ratio of mortality in cancer patients [38]. In the bivariate Cox regression analysis of this study, cancer was found to be associated with 30-day COVID-19 mortality, but in the multivariate analysis, no significance was found. It is possible that that cancer indirectly increases several parameters, such as NLR, D-Dimer, and CRP, resulting in the cancer variable not being significant during analysis.

The findings of this study have important implications for the management of COVID-19 patients in Indonesia. Healthcare professionals should be aware of the risk factors for 30-day mortality so that they can identify patients who are at high risk, and provide them with appropriate treatment. Our results are largely consistent with those of previous studies on hospitalized patients.

This study has several limitations. First, the study’s retrospective design may limit the ability to establish causal relationships from the statistical associations observed. Finally, we also did not have data of mechanical ventilation use among patients, nor detailed information on the type and dosage of steroid use among the patients.

## 5. Conclusions

This study finds that COVID-19 severity, liver cirrhosis, sex, age, leukocyte, NLR, CRP, creatinine, and procalcitonin are associated with COVID-19 mortality within 30 days. In an analysis excluding cancer patients, it was found that age, D-Dimer, CRP, and PCT were associated with 30-day mortality in COVID-19 patients, while steroid therapy is protective. These findings underscore the multifactorial nature of COVID-19 infection mortality. It is important, therefore, that patients which exhibit these factors should be treated more aggressively to prevent mortality.

## Figures and Tables

**Table 1:** Baseline characteristics.

Variables		Total (n: 644)	Expired (n: 120)	Survived (n: 524)	Comparison
Sex					0.374 ^a^
	Male	281 (43.6%)	48 (40%)	233 (44.5%)	
	Female	363 (56.4%)	72 (60%)	291 (55.5%)	
COVID-19 severity					<0.0001 ^a^
	Severe (%)	34 (5.3%)	27 (22.5%)	7 (1.3%)	
	Moderate (%)	379 (58.9%)	67 (55.8%)	312 (59.5%)	
	Mild (%)	231 (35.9%)	26 (21.7%)	205 (39.1%)	
Age (SD) (years)		48.75 ± 14.93	53.83 ± 13.98	47.57 ± 14.91	<0.0001 ^b^
BMI (SD) (kg/m^2^)		23.85 ± 4.98	22.38 ± 4.30	24.26 ± 5.05	<0.0001 ^b^
BMI					0.002 ^a^
	Obese	241 (37.4%)	28 (23.3%)	213 (40.6%)	
	Overweight	113 (17.5%)	21 (17.5%)	92 (17.6%)	
	Underweight	94 (14.6%)	25 (20.8%)	69 (13.2%)	
	Normal	196 (30.4%)	46 (38.3%)	150 (28.6%)	
Hemoglobin (SD) (g/dL)		11.54 ± 3.06	10.13 ± 2.83	11.86 ± 3.03	<0.0001 ^b^
Hematocrit (SD) (%)		35.78 ± 27.27	29.27 ± 7.84	34.51 ± 8.45	<0.0001 ^b^
Leukocyte (Min–Max) (/μL)		7980 (350–329,080)	13,180 (2170–329,080)	7360 (350–251,810)	0.068 ^c^
Platelet (Min–Max) (/µL)		249,000 (2000–842,000)	241,000 (14,000–842,000)	249,500 (2000–837,000)	0.001 ^c^
Neutrophil (Min–Max) (%)		75 (0–98.3)	84.6 (22–98.3)	71.95 (0–96.1)	0.953 ^c^
Lymphocyte (Min–Max) (%)		15.4 (3–93)	8.45 (0.3–42.6)	16.80 (1.2–93)	0.593 ^c^
NLR (Min–Max) (%)		4.87 (0–326.67)	9.91 (0.673–326.66)	4.20 (0–80.08)	0.711 ^c^
PLR (Min–Max) (%)		206.626 (0.5–3122.22)	267.7558 (1.05–3122.22)	194.7102 (0.5–2661.54)	0.095 ^c^
D-Dimer (Min–Max) (ng/mL)		960 (140–35,200)	1740 (140–35,300)	960 (160–35,200)	0.260 ^c^
CRP (Min–Max) (mg/L)		33.4 (0–2075)	83.1 (0.10–399.23)	30.8 (0–2075)	0.633 ^c^
Procalcitonin (Min–Max) (ng/mL)		0.18 (0.02–785.5)	0.71 (0.04–289.1)	0.18 (0.02–785.5)	0.879 ^c^
AST (Min–Max) (U/L)		26 (4–870)	41 (6–870)	25 (4–870)	0.033 ^c^
ALT (Min–Max) (U/L)		25 (5–2174)	26 (6–375)	25 (5–2174)	0.260 ^c^
Creatinine (Min–Max) (mg/dL)		0.9 (0.2–17)	1.1 (0.2–17)	0.8 (0.2–16.20)	0.311 ^c^
Received Steroid Therapy (%)		203 (31.5%)	52 (25.6%)	151 (74.4%)	0.004 ^a^
Cancer (%)		260 (40.4%)	78 (65%)	182 (34.7%)	<0.0001 ^a^
Liver cirrhosis (%)		10 (1.6%)	4 (3.3%)	6 (1.1%)	0.080 ^a^
Hypertension (%)		173 (26.9%)	27 (22.5%)	146 (27.9%)	0.232 ^a^
Diabetes (%)		118 (18.3%)	25 (20.8%)	93 (17.7%)	0.431 ^a^
Chronic kidney disease (%)		90 (14%)	30 (25%)	60 (11.5%)	<0.0001 ^a^
Cardiovascular disease (%)		72 (11.2%)	14 (11.7%)	58 (11.1%)	0.851

^a^ chi-square; ^b^ *t*-test; ^c^ Mann–Whitney.

**Table 2:** Bivariate and multivariate Cox regression analysis of factors associated with mortality from COVID-19 within 30 days.

		Bivariate Analysis			Multivariate Analysis		
Variables	Categories	HR	95% CI	*p* Value	HR	95% CI	*p* Value
COVID-19 Severity	Severe	4.666	2.693–8.803	<0.0001	7.024	3.871–12.744	<0.0001
	Moderate	1.533	0.974–2.413	0.065	1.660	1.048–2.629	0.031
	Mild	Reference			Reference		
Liver cirrhosis	Yes	2.452	0.900–6.684	0.080	3.422	1.208–9.691	0.021
	No	Reference			Reference		
Hypertension	Yes	0.847	0.550–1.306	0.453	-	-	-
	No	Reference					
Diabetes	Yes	1.092	0.702–1.697	0.697	-	-	-
	No	Reference					
Chronic kidney disease	Yes	1.675	1.103–2.542	0.015	-	-	-
	No	Reference					
Cardiovascular disease	Yes	1.051	0.600–1.841	0.863	-	-	-
	No	Reference					
Sex	Female	1.339	0.929–1.930	0.118	1.738	1.187–2.545	0.004
	Male	Reference			Reference		
Cancer	Yes	1.765	1.195–2.606	0.004	-	-	-
	No	Reference					
Age	≥60 years	1.468	1.013–2.128	0.043	2.139	1.279–3.577	0.004
	<60 years	Reference			Reference		
BMI	Overweight or obese	0.662	0.425–1.031	0.068	-	-	-
	Underweight	1.165	0.735–1.845	0.515			
	Normal	Reference					
Hemoglobin	Anemia	1.407	0.975–2.029	0.068	-	-	-
	No anemia	Reference					
Leukocyte	≥15,000 (/μL)	2.921	1.927–4.427	<0.0001	11.502	1.523–86.874	0.018
	<15,000 (/μL)	Reference			Reference		
Platelet	>249,000 (/μL)	1.032	0.721–1.478	0.863	-	-	-
	<249,000 (/μL)	Reference					
NLR	≥4.87	3.301	2.052–5.309	<0.0001	1.720	1.049–2.819	0.032
	<4.87	Reference			Reference		
PLR	≥206.62	1.272	0.864–1.872	0.222	-	-	-
	<206.62	Reference					
D-Dimer	≥850 (ng/mL)	1.829	1.165–2.872	0.009	1.571	0.975–2.530	0.063
	<850 (ng/mL)	Reference			Reference		
CRP	≥33.4 (mg/L)	3.436	2.047–5.768	<0.0001	1.906	1.092–3.329	0.023
	<33.4 (mg/L)	Reference			Reference		
Procalcitonin	≥0.18 (ng/mL)	4.139	2.319–7.386	<0.0001	3.281	1.780–6.049	<0.0001
	<0.18 (ng/mL)	Reference			Reference		
AST	≥130 (U/L)	2.263	1.335–3.835	0.002	-	-	-
	<130 (U/L)	Reference					
ALT	≥125 (U/L)	1.497	0.758–2.953	0.245	-	-	-
	<125 (U/L)	Reference					
Creatinine	≥0.9 (mg/dL)	1.761	1.209–2.567	0.003	1.863	1.240–2.800	0.003
	<0.9 (mg/dL)	Reference			Reference		
Received Steroid Therapy	Yes	1.017	0.703–1.472	0.929	-	-	-
	No	Reference					

**Table 3:** Results of multivariate Cox regression analysis excluding cancer patients (n: 384).

Variables	Category	HR	95% CI Lower	*p* Value
Age	≥60 years	2.225	1.184–4.182	0.013
	<60 years	Reference		
D-Dimer	≥850 (ng/mL)	2.942	1.126–7.684	0.028
	<850 (ng/mL)	Reference		
CRP	≥33.4 (mg/L)	4.356	1.665–11.398	0.003
	<33.4 (mg/L)	Reference		
PCT	≥0.18 (ng/mL)	8.098	1.913–34.278	0.004
	<0.18 (ng/mL)	Reference		
Received Steroid Therapy	Yes	0.332	0.154–0.714	0.005
	No	Reference		

## Data Availability

The data that support the findings of this study are available from the corresponding author, Ikhwan Rinaldi, upon reasonable request.
